# Residential Proximity and Symptoms of Depression and Anxiety Among Adult Children Caring for Aging Parents

**DOI:** 10.1093/geroni/igaf122.733

**Published:** 2025-12-31

**Authors:** Catherine Elmore, Soojung Ahn, Malek Alnajar, Megan Hebdon, Sarah Small, Gregory Stoddard, Katherine Ornstein

**Affiliations:** University of Utah, Salt Lake City, Utah, United States; Boston College William F. Connell School of Nursing, Chestnut Hill, Massachusetts, United States; University of Utah, Salt Lake City, Utah, United States; University of Utah College of Nursing, Salt Lake City, Utah, United States; University of Utah Department of Economics, Salt Lake City, Utah, United States; University of Utah, Study Design and Biostatistics Center, Salt Lake City, Utah, United States; Johns Hopkins University, Baltimore, Maryland, United States

## Abstract

Long-distance caregivers are an understudied subgroup of family caregivers. This study characterizes adult-child caregivers based on residential proximity to their older-adult parent, and explores associations of proximity with caregiver symptoms of depression and anxiety. Data from the 2021 National Health and Aging Trends Study Round 11 was linked with National Study of Caregiving IV. The sample included adult-child caregivers of Medicare beneficiaries aged 71 and older. Bivariate and multivariable Poisson regression models (stratified by education attainment) analyzed associations between proximity categories, caregiver demographics, care activities, and symptoms of depression and anxiety. The sample included 932 caregivers, representing a population of 9,171,811. In weighted analysis, 26.3% of caregivers coreside, 47.4% live 1–20 minutes away, 13.1% live 21–59 minutes away, and 13.2% live ≥1 hour away. Residential proximity to their aging parent was not a significant predictor of depression, and only marginally significant for anxiety in those with some college education. Poor/fair health predicted anxiety across all education groups; employment reduced the risk of depression for those with some college education. While adult-children provide varying types of caregiving assistance regardless of residential proximity, depression and anxiety are largely associated with caregivers’ health and financial status rather than residential proximity. Findings highlight the need for policies, services and programs that support the needs of distance caregivers, given that their needs are relatively overlooked compared to proximate/local caregivers.

